# Mitochondrial programmed cell death in bronchopulmonary dysplasia: mechanisms and therapeutic targets

**DOI:** 10.3389/fphys.2026.1765819

**Published:** 2026-07-15

**Authors:** Jianfeng Jiang, Mingyan Wang, Huici Yao, Ying Zhu, Hongyan Lu

**Affiliations:** Department of Pediatrics, The Affiliated Hospital of Jiangsu University, Zhenjiang, China

**Keywords:** apoptosis, bronchopulmonary dysplasia, ferroptosis, mitochondrial dysfunction, mitophagy, necroptosis, pyroptosis

## Abstract

Bronchopulmonary dysplasia (BPD) is a prevalent chronic lung disease in preterm infants, primarily characterized by arrested alveolarization and dysregulated pulmonary microvascular development. Mitochondria are crucial for normal lung development, supporting cellular energy and homeostasis. In BPD, mitochondrial homeostasis is severely disrupted, characterized by excessive fission, suppressed fusion, deficient biogenesis, imbalanced mitophagy, a collapsed antioxidant system, and a consequent energy crisis. Following mitochondrial damage, a spectrum of mitochondria-associated programmed cell death pathways is activated in pulmonary cells, including mitophagy-dependent death, apoptosis, necroptosis, pyroptosis, and ferroptosis. Within alveolar epithelial cells, vascular endothelial cells, and other pulmonary cells, these death pathways operate both independently and through extensive crosstalk, forming a complex regulatory network. This network synergistically disrupts pulmonary cell survival, proliferation, and differentiation, ultimately arresting lung development. Therapeutic strategies aim to restore mitochondrial homeostasis, inhibit specific death pathways, and utilize regenerative approaches like exosome-based delivery. This review examines the role of mitochondrial dysfunction and interconnected cell death pathways in BPD pathogenesis and discusses emerging treatments.

## 1 Introduction

Bronchopulmonary dysplasia (BPD) is a common chronic lung disease in preterm infants, particularly those born extremely premature, characterized by arrested alveolarization and disrupted pulmonary microvascular development (Thebaud et al., 2019). Current research indicates that BPD is a multifactorial disease. Prematurity and lung immaturity serve as the baseline, upon which adverse factors such as mechanical ventilation, oxygen toxicity, and infection collectively hinder lung development. A major risk factor is the excessive production of reactive oxygen species (ROS) in a hyperoxic environment (Shukla and Ambalavanan, 2021). Excessive ROS induces injury and death of type II alveolar epithelial cells (AEC II) and pulmonary vascular endothelial cells (PVEC), resulting in alveolar simplification, vascular leakage, and inflammatory cell recruitment, ultimately leading to arrested alveolar-capillary development (Wang and Dong, 2018).

Importantly, mitochondria are the primary intracellular source of hyperoxia-induced ROS and function as central regulators of cellular metabolism, signaling, and fate determination (Nunnari and Suomalainen, 2012). Under stress conditions, mitochondrial homeostasis is disrupted, leading to excessive mtROS production, bioenergetic failure, and collapse of mitochondrial quality control (mtQC). This dysfunction triggers a cascade of mitochondrial injury events that extend beyond energy deficiency to activate multiple programmed cell death (PCD) pathways. Mitochondria serve as key regulators of intrinsic apoptosis via cytochrome c release and BCL-2 family–mediated mitochondrial outer membrane permeabilization program (Czabotar and Garcia-Saez, 2023). In addition, mitochondrial damage amplifies pyroptosis, necroptosis, and ferroptosis through release of mitochondrial danger signals such as mtDNA, cardiolipin, and ROS, thereby linking metabolic dysfunction with inflammatory cell death signaling (Flores-Romero et al., 2023). These interconnected pathways establish mitochondria as a central hub integrating oxidative stress, metabolic collapse, and inflammatory injury in BPD.

In preterm lungs exposed to hyperoxia, excessive mtROS disrupts mitochondrial quality control systems, including mitophagy, fission–fusion balance, and biogenesis, thereby driving progressive cellular energy failure (Ratner et al., 2009). Impaired mtQC sensitizes pulmonary cells to autophagy-dependent cell death and ferroptosis, while sustained stress further shifts cell fate toward apoptosis and inflammatory pyroptosis signaling (Chen et al., 2024; Saller et al., 2025) program toward. These cell death pathways, in turn, converge on mitochondria as a signaling hub, interacting with one another across alveolar epithelial cells, vascular endothelial cells, and pulmonary interstitial cells, collectively contributing to arrested lung development and tissue remodeling in BPD (Deng et al., 2022b). Currently, no therapies can reverse this developmental arrest. Therefore, elucidating the role of mitochondrial dysfunction and PCD networks in BPD is essential for identifying effective mechanistic therapeutic strategies. Therefore, this article first focuses on the shifting role of mitochondria from developmental homeostasis to developmental impairment, followed by a review of mitochondrial damage, the mitochondria-associated PCD pathways it triggers in BPD, and the corresponding treatment strategies.

## 2 The role of mitochondria in lung development

During normal lung development, the proliferation and differentiation of lung tissue cells are highly energy-demanding processes. AEC II serve as the stem cells of the alveolar epithelium, with their proliferation and differentiation central to alveolar development, while PVEC are the fundamental cells constructing the pulmonary vasculature. These developmental processes are highly dependent on mitochondrial oxidative phosphorylation, which provides ATP to support rapid cell growth and tissue remodeling (Schittny, 2017). Importantly, mitochondrial function is particularly critical during the alveolarization window, as epithelial cells exhibit high sensitivity to mitochondrial perturbations. For example, specific knockout of the mitochondrial complex I subunit Ndufs2 in lung epithelial cells leads to severe alveolar developmental arrest and postnatal lethality in neonatal mice, demonstrating the indispensable role of mitochondrial energy metabolism in lung development modeling (Han et al., 2023). Mitochondria not only provide cellular energy, but also regulate lung cell fate through metabolic control of differentiation. For instance, altered mitochondrial metabolism has been associated with impaired alveolar epithelial maturation and disrupted epithelial–mesenchymal signaling during lung development, accompanied by a developmental metabolic shift from glycolysis-dominant early stages toward increased mitochondrial oxidative phosphorylation during later alveolarization program (Li et al., 2025).

Mitochondria also determine the fate of lung tissue cells by dynamically regulating ATP production, ROS balance, and cellular signaling transduction. Research indicates that reduced mitochondrial abundance and altered mitochondrial subcellular localization in AEC II decrease the production of ligands such as platelet-derived growth factor (PDGF) in these cells, thereby hindering the formation of the alveolar structure by myofibroblasts (Zhang et al., 2022). During the peak postnatal period of alveolarization, deficiency of mitochondrial respiratory chain complex I (e.g., the NDUFS2 subunit) in AEC II activates the ISR, thereby inhibiting the translation of key transcription factors essential for alveolar development. This subsequently suppresses the proliferation and differentiation program of alveolar epithelial cells, ultimately leading to arrested alveolar development (Han et al., 2023). Conversely, mitochondrial dysfunction induced by disruption of matrix proteostasis, such as LONP1 deficiency, further highlights the cell-type-specific vulnerability of airway and alveolar epithelial cells, where stress responses including ISR activation can drive apoptosis or impair regenerative capacity while (Xu et al., 2024). Collectively, these findings demonstrate that mitochondria orchestrate lung development not only through energy supply, but also through precise regulation of epithelial–vascular signaling and stress-response pathways that determine cellular fate during alveolar and vascular formation.

## 3 Impact of mitochondrial damage on BPD pathogenesis

**Figure 1:**
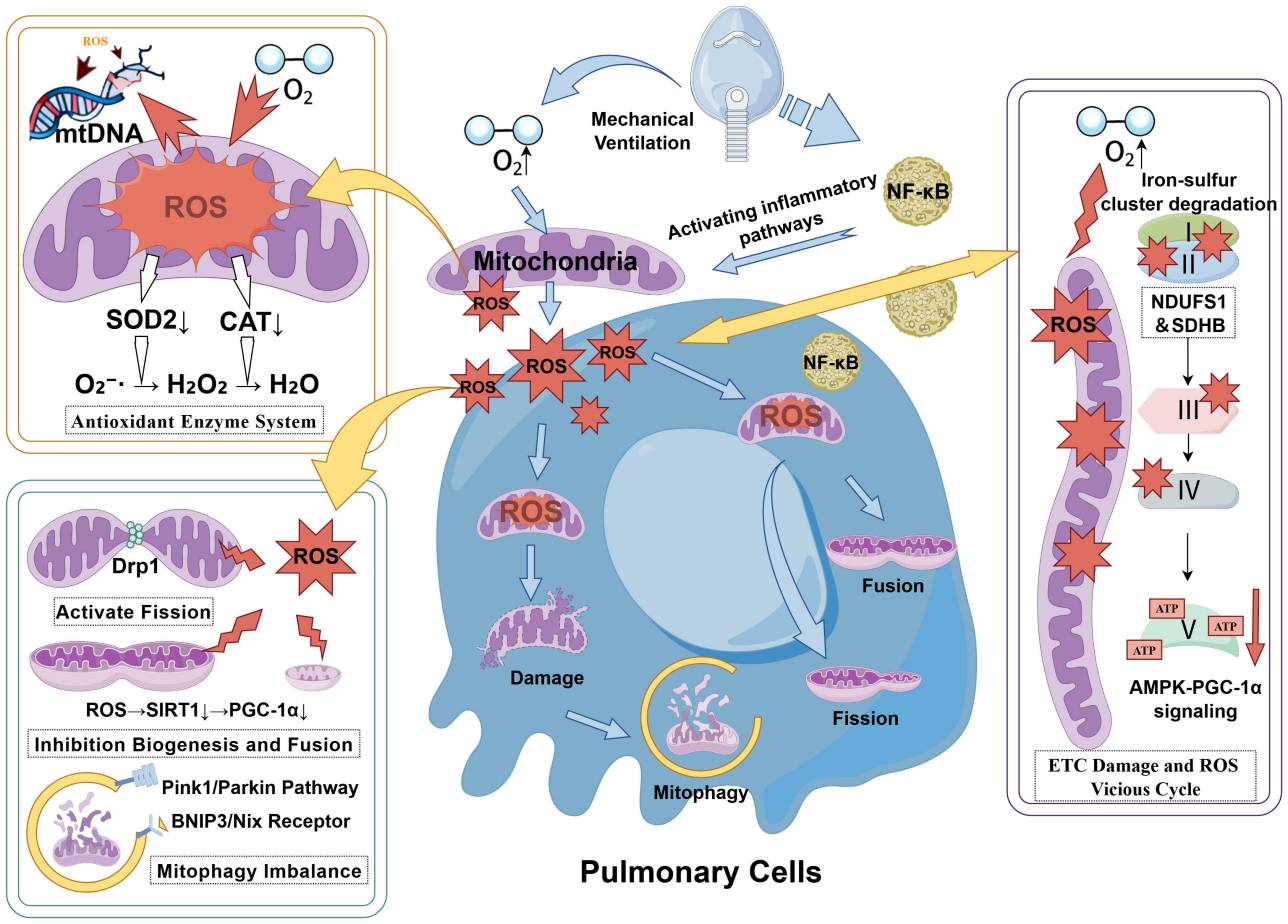
The vicious cycle of mitochondrial damage in BPD. This diagram illustrates the core vicious cycle through which mechanical ventilation and hyperoxia induce mitochondrial damage in pulmonary cells by triggering excessive ROS production: Firstly, ROS directly attack mitochondria and suppress the antioxidant defense system (SOD2/CAT). Secondly, ROS disrupt mtQC by impairing fission (via Drp1) and mitophagy (via the PINK1/Parkin pathway). Concurrently, ROS inflict direct damage on the ETC, leading to ATP synthesis failure and an energy crisis. Dysfunctional mitochondria in turn generate more ROS, forming a self-reinforcing “ROS-ETC damage” loop that perpetuates the pathological progression of BPD.

While mitochondria play a precisely regulated role in physiological lung development, their homeostasis is highly vulnerable to disruption when lung tissue is exposed to pathological insults such as hyperoxia, mechanical stretch, or inflammation before or after birth. Such disruption leads to mitochondrial damage, including rupture of the mitochondrial membrane, mtDNA fragmentation, and imbalance in mtQC. This damage subsequently triggers mitochondria-associated PCD, diverting the lung from a state of developmental homeostasis towards the pathological outcome of BPD (**Figure 1**).

​ Functional impairment of the mitochondrial antioxidant enzyme system serves as the initial trigger for mitochondrial damage in BPD. Mitochondria possess intrinsic enzymatic defenses against ROS, but preterm infants exhibit immature antioxidant capacity, which is further overwhelmed by hyperoxia-induced ROS production. Key antioxidant enzymes, including superoxide dismutase 2 (SOD2) and catalase (CAT), are significantly reduced in preterm lung tissues, while downregulation of the mitochondrial regulator PGC-1α further exacerbates oxidative vulnerability defense (Cui et al., 2019; Mohammadi et al., 2022). Excessive mtROS promotes lipid peroxidation of mitochondrial membranes rich in polyunsaturated fatty acids, leading to structural damage and mtDNA injury (M Rüdiger et al., 2000). Together, oxidative enzyme deficiency and ROS overproduction disrupt mitochondrial integrity, ultimately leading to the failure of mtQC.

Imbalance in mtQC represents the most critical juncture in mitochondrial dysfunction during BPD. Hyperoxia and inflammatory stress induce excessive mitochondrial fission in lung cells, primarily mediated by the Drp1 pathway, leading to mitochondrial fragmentation, metabolic reprogramming, and reduced ATP production program (Dai et al., 2021; Sun et al., 2024). Conversely, mitochondrial fusion and biogenesis are suppressed through dysregulation of the SIRT1–SUMO1 axis and downregulation of fusion-related proteins such as Mfn1/2, resulting in impaired mitochondrial network integrity and reduced bioenergetic capacity (Yang et al., 2025). Mitophagy is also disrupted in BPD. Hyperoxic stress activates the PINK1/Parkin and BNIP3/NIX pathways, but sustained activation leads to excessive or dysregulated mitochondrial clearance, further aggravating energy depletion and cellular stress (Wu et al., 2019; Yu et al., 2020). In addition, impaired mitochondrial biogenesis may facilitate intercellular mitochondrial transfer from mesenchymal stem cells to injured epithelial cells, suggesting a compensatory but dysregulated response within the mitochondrial network (Huang et al., 2023). Collectively, mtQC imbalance disrupts mitochondrial homeostasis, leading to energy failure and contributing to impaired lung development in BPD.

Oxidative damage to the ETC complexes, leading to ATP deficiency, serves as the direct driver of the energy crisis in BPD. Hyperoxia destabilizes iron–sulfur (Fe–S) cluster–containing proteins and impairs key respiratory chain complexes, particularly complex I (NDUFS1) and complex II (SDHB), leading to reduced mitochondrial oxygen consumption and impaired electron transport sulfur (Baik et al., 2023). This damage is observed not only in cell models exposed to hyperoxia but has also been verified in BPD mouse models and lung tissue samples from preterm infants. Hyperoxia suppresses the AMPK-PGC−1α signaling axis mentioned above, which reduces ETC complex activity and ATP production (Rana et al., 2024), Moreover, the collapse of mitochondrial membrane potential and impaired calcium handling further exacerbate bioenergetic failure (Ten and Ratner, 2020).

In summary, mitochondrial oxidative damage and bioenergetic failure synergistically drive cellular dysfunction in BPD. Impaired antioxidant defense, excessive mtROS production, and ETC dysfunction collectively disrupt mitochondrial integrity and energy homeostasis, rendering pulmonary epithelial and endothelial cells highly susceptible to programmed cell death and contributing to arrested lung development defense.

## 4 Impact of mitochondria-associated PCD in pulmonary cells on BPD pathogenesis

When exposed to triggers of BPD such as hyperoxia, inflammation, and mechanical ventilation, pulmonary cells undergo mitochondrial structural damage and loss of quality control, leading to energy deficiency and amplified oxidative stress. These functional disturbances further activate PCD mechanisms in pulmonary cells, including mitophagy-dependent cell death, apoptosis, pyroptosis, necroptosis, and ferroptosis. While these forms of PCD operate through distinct mechanisms, they are interrelated, sharing mitochondrial damage as a common upstream trigger. Within alveolar epithelial cells, vascular endothelial cells, and other pulmonary cells, they form a complex signaling network that collectively drives a vicious cycle of mitochondrial failure, sustained inflammation, and arrested lung development. In the following sections, we systematically summarize how mitochondria-associated cell death pathways in different pulmonary cell types contribute to the development of BPD.

### 4.1 Impact of mitochondria-associated PCD in AEC II on BPD pathogenesis

The alveoli serve as the primary site for gas exchange, and AEC II are central to alveolar development because they function as epithelial progenitors, secrete pulmonary surfactant, and differentiate into AEC I during lung maturation and repair (Rodriguez-Castillo et al., 2018; Aspal and Zemans, 2020). Current evidence indicates that mitochondrial damage in BPD further induces various forms of mitochondria-associated PCD in AEC II, including mitophagy-dependent cell death, apoptosis, pyroptosis, necroptosis, and ferroptosis. This ultimately leads to classic BPD pathological features such as reduced alveolar number and thickened alveolar septa.

Mitophagy exhibits a dual regulatory role in AEC II. The core pathway governing mitophagy is the PINK1-Parkin pathway: when mitochondrial membrane potential declines, PINK1 stabilizes on the mitochondria and recruits Parkin, which ubiquitinates mitochondrial substrates. The ubiquitinated cargo is then recognized by the adaptor protein p62, which links it to LC3, thereby guiding autophagosome encapsulation and degradation of the damaged mitochondria (Yamada et al., 2025). Additionally, the NIX receptor pathway can directly initiate mitophagy (Yu et al., 2020; Onishi et al., 2021). In hyperoxia-induced BPD models, enhancing autophagic flux with the mTOR inhibitor Torin2 increases LC3 conversion and p62 degradation, reduces cleaved caspase-3 and the Bax/Bcl-2 ratio, and improves alveolarization, suggesting that appropriately activated mitophagy protects AEC II from mitochondrial apoptosis (Sureshbabu et al., 2016). Similarly, itaconate promotes TFEB nuclear translocation, enhances lysosomal biogenesis and autophagic flux, increases SP-C expression, and reduces AEC II death in BPD models (Zhang et al., 2018; Liu et al., 2024a). Meanwhile, other studies indicate that under sustained hyperoxia, excessive mitophagy may promote cell death by compromising mtQC, whereas downregulation of autophagy−related proteins promotes inflammation resolution, thereby preventing autophagy-dependent cell death in AEC II (Wu et al., 2019; Nah et al., 2020). Thus, the key pathological issue in BPD is not simply insufficient or excessive mitophagy, but the loss of mitophagic balance that determines whether AEC II undergo repair or progress toward mitophagy-dependent cell death.

Apoptosis is one of the most extensively studied forms of cell death in AEC II in BPD and is closely linked to impaired alveolarization. Mitochondrial apoptosis is initiated when damaged mitochondria activate BH3-only proteins and shift the Bcl-2 family balance toward Bax/Bak-dependent mitochondrial outer membrane permeabilization (MOMP), followed by Cytc release, apoptosome formation, and caspase-9/3/6/7 activation (Adams and C.S., 2001; Glover et al., 2024). In BPD, apoptosis in AEC II not only follows a precisely regulated cell death program but also involves modulation by complex signaling networks. Studies indicate that stressors such as hyperoxia and inflammation disrupt the homeostasis of the SIRT1–SENP1 axis, thereby affecting the balance of SUMOylation/de−SUMOylation modifications on key proteins and ultimately inducing mitochondrial apoptosis in AEC II (Dong et al., 2021; Zhang et al., 2025). Inhibition of PI3K/AKT signaling promotes FoxO3a nuclear translocation and Bim expression, thereby enhancing mitochondrial apoptosis in AEC II signaling (Wu et al., 2018). Furthermore, abnormal ERK/SMAD3 activity, Notch pathway dysregulation, and persistent inflammatory NF-κB signaling further amplify apoptotic susceptibility under hyperoxic conditions signaling (Jiang et al., 2021; Liu et al., 2024b). Insufficient activation of the Nrf2 antioxidant pathway also increases ROS burden, thereby lowering the threshold for MOMP and caspase activation (Amata et al., 2017; Ma et al., 2020). These findings indicate that AEC II apoptosis in BPD is driven by convergence of oxidative stress, impaired survival signaling, and mitochondrial membrane destabilization, ultimately depleting the epithelial progenitor pool required for alveolar repair.

Pyroptosis, a highly inflammatory form of PCD, is closely linked to the imbalance of mtQC. In AEC II during BPD, mitochondria-associated pyroptosis is initiated when oxidative stress is sensed by the NLRP3 inflammasome, which recruits caspase−1 to cleave Gasdermin D (GSDMD), thereby forming membrane pores. This leads to the release of IL−1β/IL−18, resulting in cell lysis and a potent inflammatory response (Cookson and Brennan, 2001; Kesavardhana et al., 2020). BPD-specific studies further suggest that pyroptosis is tightly connected with impaired autophagy rather than acting as an isolated pathway. METTL3-mediated m6A methylation can suppress the mitophagy-related gene ATG8, disrupt ATG8–GSDMD interaction, block autophagic flux, and activate the caspase-1/GSDMD axis, thereby promoting inflammatory AEC II pyroptosis under hyperoxic conditions. towards (Xu et al., 2023). Conversely, rapamycin-mediated activation of mTOR-dependent autophagy suppresses NLRP3 and caspase-11 inflammasome activation, reduces IL-1β and IL-18 release, and improves alveolar simplification in experimental BPD (Zhang et al., 2023). Therefore, pyroptosis in AEC II could be considered as a BPD-relevant consequence of mitochondrial damage combined with defective autophagic clearance, which converts epithelial injury into an inflammatory amplification loop.

Ferroptosis, an iron-dependent form of PCD, also impacts the survival and development of AEC II. During the progression of BPD, hyperoxic stimulation leads to iron overload (Fe²^+^), which in turn generates a large amount of ROS via the Fenton reaction. This ROS attacks polyunsaturated fatty acids (PUFAs) in the mitochondrial membrane lipids, initiating a lipid peroxidation chain reaction that accelerates the collapse of mitochondrial membrane structure (Jelinek et al., 2018; Stockwell et al., 2020). Hyperoxia-induced bursts of mtROS and damage to the ETC not only directly trigger lipid peroxidation but also inactivate glutathione peroxidase 4 (GPX4) and deplete glutathione (GSH). This failure of the antioxidant defense system consequently establishes a microenvironment conducive to ferroptosis (Yang et al., 2023a). Experiments have confirmed that in the lungs of hyperoxia-exposed neonatal mice, levels of Fe²^+^, malondialdehyde, and iron deposition are significantly elevated, while GPX4 activity and the degree of alveolarization are markedly reduced. Administration of a ferroptosis inhibitor effectively alleviated AEC II injury and promoted alveolar repair (Ruan et al., 2025). Thus, ferroptosis provides a mechanistic bridge between mitochondrial oxidative injury, membrane lipid damage, and loss of epithelial regenerative capacity in BPD.

Necroptosis is activated as a backup cell death mechanism when the apoptotic pathway is inhibited, ensuring that cells can still execute a death program via the RIPK3/MLKL axis even when caspase−8−mediated extrinsic apoptosis is blocked. Specifically, ROS initially induces the deubiquitination of RIPK1, which then forms the necrosome with RIPK3. This complex subsequently phosphorylates MLKL, disrupting plasma membrane integrity and ultimately leading to cell swelling, necrosis, release of DAMPs, and an inflammatory response (Ye et al., 2023). Studies have confirmed that hyperoxia exposure significantly upregulates the expression of RIP1, RIP3, and MLKL in lung tissue, which is accompanied by an increase in necroptosis of AEC II. Both the free radical scavenger edaravone and the specific necroptosis inhibitor Nec−1 can ameliorate alveolar structural disorganization and pulmonary edema by suppressing this pathway (Han et al., 2018). These results demonstrate that in BPD, when apoptosis is impaired, necroptosis acts as a complementary cell−death mechanism that cooperatively promotes AEC II cell death and impedes alveolar development.

### 4.2 Impact of mitochondria-associated PCD in PVEC on BPD pathogenesis

PVEC are central cells in the pathogenesis of pulmonary vascular dysregulation in BPD. They form the endothelial component of the alveolar-capillary barrier and are essential for pulmonary microvascular development. Beyond this structural role, injured PVEC actively participate in inflammation by expressing adhesion molecules and releasing inflammatory mediators that promote leukocyte recruitment (Pokharel et al., 2023). In BPD, apoptosis, inflammation, and pyroptosis in PVEC are closely interconnected. Together, they lead to disordered pulmonary microvascular development and impaired microcirculation, which subsequently induces microvascular remodeling and contributes to the development of pulmonary hypertension.

Numerous studies indicate that mitochondria−mediated intrinsic apoptosis is also the predominant mode of cell death in PVEC during BPD. This process is coordinately regulated by multiple signaling pathways, including Notch, NF−κB, and ERK, which collectively contribute to the developmental impairment of the alveolar−vascular unit. The Notch pathway, a key regulator of angiogenesis, shows dysregulated expression closely associated with alveolar simplification (Zhan et al., 2021). Under hyperoxic conditions, decreased expression of the mitochondrial function regulator LKB1 can induce excessive activation of Notch signaling and upregulation of downstream targets such as Hes1, thereby promoting endothelial apoptosis and defective angiogenesis (Deng et al., 2022a; Rana et al., 2024). The NF−κB pathway exhibits sustained activation in BPD. While initially conferring some protective effects, its prolonged activation leads to the massive release of inflammatory cytokines such as TNF−α and IL−1β, which promote pro-apoptotic mediators including Bim/Bax while weakening survival proteins Bcl-2/Bcl-xL while, thereby amplifying endothelial cell apoptosis (Iosef et al., 2012; McKenna et al., 2014). Under sustained hyperoxic stress, abnormal ERK activation may also induce p53/p21-mediated cell-cycle arrest and metabolic dysregulation, thereby indirectly activating the mitochondrial apoptotic pathway signaling (Menon et al., 2018, 2022). In summary, Notch dysregulation, NF-κB-mediated inflammation, and ERK-related growth arrest converge on mitochondrial apoptosis in PVEC, ultimately impairing angiogenesis and alveolar-capillary formation. signaling while program.

Pyroptosis in PVEC is another important mitochondria-associated PCD pathway contributing to vascular dysfunction in BPD. Mitochondrial damage releases mtROS and mtDNA, which act as DAMPs to activate the NLRP3 inflammasome (Zheng et al., 2022). Inflammasome activation subsequently triggers caspase-1, or caspase-4/5 in human endothelial cells, leading to GSDMD cleavage, membrane pore formation, cell swelling, and lytic endothelial death (Cheng et al., 2017). Simultaneously, caspase−1 promotes maturation and release of IL-1β and IL-18 through GSDMD pores, further recruiting inflammatory cells and amplifying local lung inflammation (Nadeau-Vallee et al., 2017). Consequently, PVEC pyroptosis not only causes endothelial barrier disruption, increased vascular permeability, and pulmonary edema, but also impairs vascularization and may propagate inflammatory signals through extracellular vesicles or inflammasome-related mediators (Wu et al., 2022; Young et al., 2024). Mitochondrial damage-induced pyroptosis in PVEC therefore represents a key link between endothelial injury, persistent inflammation, and pulmonary vascular developmental arrest in BPD.

### 4.3 Impact of mitochondria-associated PCD in other pulmonary cells on BPD pathogenesis

The alveolar septa, which separate adjacent alveoli, contain other essential pulmonary cells. Among these, pulmonary macrophages orchestrate the immune response by phagocytosing pathogens and cellular debris and by secreting inflammatory mediators. Fibroblasts, on the other hand, synthesize the extracellular matrix to maintain alveolar structural stability, and their abnormal activation is closely linked to pathological processes such as pulmonary fibrosis and chronic inflammation (Meng et al., 2023). In BPD, tissue-resident immune cells like macrophages are involved in pulmonary immune and inflammatory reactions. Furthermore, mitochondrial damage regulates the aberrant differentiation of fibroblasts into myofibroblasts, thereby exacerbating the pathological progression of interstitial fibrosis in BPD.

Pulmonary macrophages recognize mitochondria-derived DAMPs and activate inflammatory and redox-sensitive pathways. The Nrf2 pathway is a key antioxidant defense mechanism, and myeloid-specific Nrf2 deficiency aggravates hyperoxia-induced alveolar simplification and inflammation, accompanied by reduced expression of antioxidant genes (Hmox1, Nqo1, and Gclc) and increased IL-1β levels recognize signaling defense (Tamatam et al., 2024). On the other hand, the inflammatory microenvironment driven by mitochondrial dysfunction promotes significant upregulation of secreted phosphoprotein 1 (SPP1) in macrophages. SPP1 then interacts with fibroblasts, activating the PI3K-AKT and ERK/MAPK pathways, thereby amplifying pulmonary inflammation and fibrotic responses (Liu et al., 2024b). Furthermore, mitochondrial metabolic reprogramming contributes to macrophage polarization imbalance, and vitamin A or D treatment alleviates BPD-like injury by restoring the M1/M2 balance toward (Zhen et al., 2021). Beyond macrophages, invariant natural killer T cells may exacerbate BPD by promoting ferroptosis-related injury in AEC II, involving GPX4, FTH1, and PTGS2 (Wang et al., 2024). Thus, these immune cells contribute to BPD progression through mitochondrial oxidative stress, inflammatory signaling, and impaired epithelial repair.

Pulmonary interstitial fibroblasts, serving as crucial structural support cells for alveolar architecture, are highly sensitive to mitochondrial energy disturbance and oxidative stress. Hyperoxia-induced mitochondrial dysfunction can drive fibroblast activation and aberrant differentiation into myofibroblasts, contributing to alveolar simplification and fibrotic remodeling. At the level of cell death, necroptosis emerges as a significant terminal pathway for fibroblasts. When the apoptotic pathway is inhibited, stimuli such as TNF−α trigger necroptosis via the RIPK1/RIPK3/MLKL axis, a process that is also highly dependent on the massive generation of mtROS (Hussain et al., 2018). Meanwhile, epithelial-derived IL-11 promotes fibroblast-to-myofibroblast differentiation through ERK signaling, increasing α-SMA and Collagen I expression, while IL-11 blockade alleviates hyperoxia-induced pulmonary fibrosis in neonatal mice signaling (Zhu et al., 2024). In BPD, fibroblasts function as both metabolic sensors and effector cells: mitochondrial oxidative stress promotes their death or activation, while abnormal extracellular matrix deposition and myofibroblast expansion further disrupt epithelial-endothelial homeostasis signaling while modeling (El Agha and Thannickal, 2023).

## 5 Crosstalk and mechanisms of mitochondria-associated PCD in BPD

**Figure 2:**
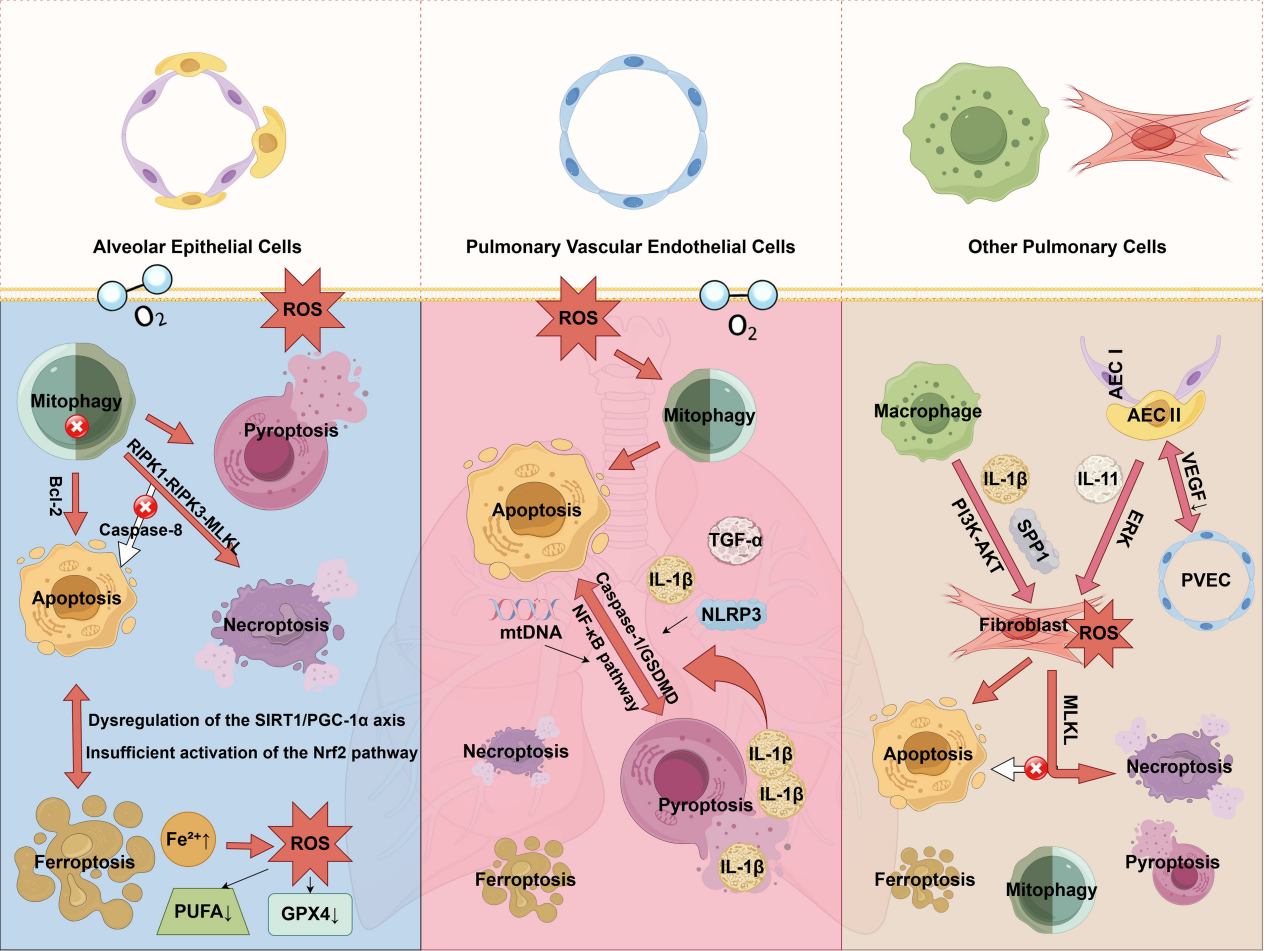
Mitochondria-associated PCD in BPD. This diagram illustrates the multi−cellular interactive network of PCD triggered by mitochondrial damage and ROS in BPD. In AEC II, impaired mitophagy can induce apoptosis and promote a ferroptosis−prone microenvironment, while also contributing to pyroptosis. Additionally, intrinsic apoptosis and necroptosis can dynamically switch via caspase−8. In vascular endothelial cells, damage−associated signals released during apoptosis are amplified into pyroptosis through NLRP3 inflammasome activation. Meanwhile, macrophages and epithelial cells drive fibroblast activation and fibrosis by secreting cytokines such as IL−1β and IL−11, collectively forming a trans−cellular network of death signaling.

In BPD, the demise of cells such as AEC II and PVEC is driven by distinct PCD pathways, underscoring their critical role in the disease’s pathology. As summarized previously, various PCD pathways, including autophagy−dependent death, apoptosis, necroptosis, pyroptosis, and ferroptosis, are involved in BPD. Each engages different molecular and cellular processes, leading to distinct morphological and functional outcomes. A growing body of evidence now indicates that these PCD pathways do not operate in isolation; rather, they engage in complex crosstalk within the context of BPD, with their underlying mechanisms and regulatory circuits extensively intersecting. For instance, caspase-1/11–GSDMD activation links inflammasome signaling to pyroptotic injury, whereas caspase-8 may function as a molecular switch between apoptosis and RIPK1/RIPK3/MLKL-mediated necroptosis while signaling. Therefore, after systematically outlining the mechanisms of different PCD types in pulmonary cells during BPD, we aim to consider diverse PCD modalities as components of a coordinated cell−death system and will explore the potential interactions between these pathways (**Figure 2**).

Current evidence indicates that mitochondrial damage and the resulting loss of quality control initially induce apoptosis in AEC II. The autophagy−enhancing peptide Tat−Beclin1 can significantly reduce hyperoxia−induced apoptosis and improve lung architecture by activating autophagic flux (Zhou et al., 2023). Impaired autophagic flux may remove the restraint on NLRP3/caspase-1/11–GSDMD signaling, thereby shifting epithelial injury from apoptosis-dominant death toward inflammatory pyroptosis toward (Zhang et al., 2023). Mitochondrial apoptosis and incomplete clearance of injured cells can compromise endothelial integrity and promote the extracellular accumulation of mitochondrial danger signals, including mtROS- and mtDNA-related signals (Shahzad et al., 2022). Furthermore, apoptosis and necroptosis can undergo dynamic switching of cell death signals through the molecular switch caspase-8. When caspase-8 is inhibited, ROS acts as a common mediator that also promotes MLKL phosphorylation, shifting the cell towards RIPK3/MLKL-dependent necroptosis. This interplay reflects the complexity and redundancy inherent in the cell death regulatory network.

There is also an interaction and transformation between endothelial cell apoptosis and pyroptosis in BPD. Endothelial injury-associated TNF-α and IL-1β amplify NF-κB signaling and further activate the NLRP3/caspase-1/GSDMD axis in neighboring endothelial or epithelial cells signaling (Zeng et al., 2024). Pyroptotic cells, in turn, release large quantities of inflammatory mediators through GSDMD pores, establishing a “apoptosis-inflammation-pyroptosis” positive feedback loop. At the histological level, this manifests as loss of vascular barrier function, leading to increased permeability and tissue edema, which hinders alveolar−vascular co−development and may also impair alveolar epithelial function via inflammatory signaling (Young et al., 2024).

Ferroptosis also exhibits profound crosstalk with other forms of cell death. In hyperoxia-induced BPD, ETC dysfunction and mtROS accumulation promote iron-dependent lipid peroxidation, reflected by increased Fe²^+^, MDA and iron deposition, together with reduced GPX4 activity and GSH-dependent antioxidant capacity. These changes sensitize AEC II to ferroptosis and may further lower the threshold for mitochondrial apoptosis. Dysregulation of the SIRT1/PGC−1α axis, coupled with increased ROS derived from NOX4, collectively exacerbates oxidative stress (Yang et al., 2023b). Meanwhile, insufficient activation of the Nrf2 pathway impairs the cell’s defense against ferroptosis, predisposing pulmonary cells to either apoptotic or ferroptotic outcomes (Yang et al., 2023a).

In summary, mitochondria and their associated PCD pathways play a central driving role in the initiation and progression of BPD. When the developing lung is exposed to injurious environments such as hyperoxia, its finely tuned homeostatic regulation is disrupted. The ensuing damage, including compromised mitochondrial membrane integrity, mtDNA fragmentation, and imbalances in mtQC such as fission/fusion, biogenesis, and mitophagic clearance, collectively constitutes the starting point of cell demise. This series of mitochondrial injuries triggers an mtROS storm and an energy crisis, which in turn activates an intricate network of cell death. This network exhibits complex crosstalk among alveolar epithelial cells, vascular endothelial cells, and pulmonary interstitial cells, collectively driving the pathological progression of BPD. Mechanistically, this network is organized around several switch-like nodes, including mitophagy/autophagic flux, caspase-8-dependent apoptosis–necroptosis switching, NLRP3–GSDMD-mediated inflammatory death, and Nrf2/GPX4-regulated resistance to lipid peroxidation. Ultimately, this cascade network, originating from mitochondrial damage and structured by the crosstalk of death pathways, collectively leads to the failure of AEC II, disruption of the PVEC barrier, and dysregulation of the interstitial microenvironment and ultimately lead to BPD.

## 6 Targeted therapeutic strategies

Given the crucial role of mitochondria and their associated PCD pathways in BPD, therapeutic strategies can be broadly summarized into three categories: restoration of mtQC and redox homeostasis, inhibition of key mitochondria-associated PCD pathways, and MSC/exosome-based regenerative therapy program (**Table 1**).

**Table 1:** Targeted therapy strategy in BPD.

Intervention level	Therapeutic strategy	Representative agents/methods	Key references
​Mitochondrial quality control system​			
Mitochondrial Dynamics Regulation	Inhibit Mitochondrial Excessive Fission	Mdivi-1	Sun et al., 2024
	Promote Mitochondrial Fusion	S89	Guo et al., 2023
Biogenesis Enhancement	Enhance Mitochondrial Biogenesis	Resveratrol	Wang et al., 2020
Redox Homeostasis	Targeted Antioxidant Therapy	MitoQ	Jelinek et al., 2018
	Activate Endogenous Defense	Nrf2 Agonists	Weng et al., 2021
Programmed cell death pathway intervention​			
Autophagy Modulation	Stage-Specific Regulation	Melatonin	Deng et al., 2025
Apoptosis Inhibition	Multi-Target Synergistic Inhibition	Rapamycin; Metformin	Yang et al., 2025;
Inhibition of Other PCD	Inhibit Necroptosis	Necrostatin-1	Han et al., 2018
	Inhibit Pyroptosis	Caffeine	Dapaah et al., 2019
	Inhibit Ferroptosis	​Ferrostatin−1	Ruan et al., 2025
​Cell replacement and regenerative therapy​			
​MSC Therapy	Immunomodulation	MSC-derived Exosomes	Willis et al., 2018
	Promote Tissue Repair	MSC-derived Exosomes	Zhang et al., 2023
	Mitochondrial Transfer	MSC-based Transplantation	Omar et al., 2022

For mtQC restoration, suppressing excessive Drp1-mediated mitochondrial fission remains a key target, as mitochondrial fragmentation contributes to alveolar epithelial apoptosis and microvascular endothelial dysfunction; Mdivi-1 has been shown to improve mitochondrial network integrity, enhance energy metabolism, and reduce oxidative injury in pulmonary cells (Dai et al., 2021; Sun et al., 2024). In parallel, promoting mitochondrial fusion and biogenesis through targets such as MFN2 or the SIRT1-PGC-1α-NRF1 axis may help restore mitochondrial membrane potential, ATP synthesis, and respiratory chain assembly (Wang et al., 2020; Ru et al., 2023). Because antioxidant failure is a major upstream event in BPD-related mitochondrial injury, mitochondria-targeted antioxidants such as MitoQ and activation of endogenous Nrf2-dependent defense pathways may further reduce mtROS accumulation and protect alveolar epithelial and vascular endothelial function (Jelinek et al., 2018) (Chu et al., 2020; Weng et al., 2021). These multimodal strategies, by coordinately regulating the mtQC system and the antioxidant enzyme system, provide a foundational basis for combined interventions aimed at repairing the developmental impairment of the alveolar-capillary unit in BPD.

Inhibiting mitochondria-associated PCD represents the second therapeutic direction, but the intervention should be pathway- and stage-specific rather than a simple blockade of cell death. For mitophagy, early enhancement of impaired autophagic flux may promote clearance of dysfunctional mitochondria, whereas excessive activation at later stages may require restoration of mitophagic balance, including regulation of the PINK1/Parkin or BNIP3L-related pathways (Wu et al., 2019; Deng et al., 2025). For apoptosis, agents such as rapamycin, SENP1-targeted approaches, resveratrol, and metformin may act through autophagy enhancement, SIRT-related mitochondrial protection, AMPK-PGC-1α-mediated biogenesis, Nrf2 activation, and inhibition of Notch/NF-κB inflammatory signaling (Chu et al., 2020; Yadav et al., 2020; Zhan et al., 2021). For other forms of PCD, specific inhibitors show considerable therapeutic promise. Necroptosis can be blocked by Nec−1, which inhibits the RIPK1−RIPK3−MLKL axis (Han et al., 2018);Pyroptosis inhibition targets the NLRP inflammasome−GSDMD pathway, as exemplified by caffeine suppressing NLRP3 assembly and IL−1β release (Dapaah-Siakwan et al., 2019; Chen et al., 2020);Intervention in ferroptosis focuses on restoring GPX4 activity and inhibiting lipid peroxidation to suppress the ferroptosis−prone microenvironment (Jelinek et al., 2018; Yang et al., 2023a; Ruan et al., 2025).

Mesenchymal stem cells (MSCs) and their exosomes represent a regenerative strategy that may act through immunomodulation, tissue repair, and directional mitochondrial transfer. MSC-derived exosomes can be taken up by alveolar macrophages and promote macrophage polarization away from the pro-inflammatory M1 phenotype toward a reparative M2 phenotype, thereby reducing hyperoxia-induced pulmonary inflammation (Willis et al., 2018). More importantly, there is a directional process in mitochondrial transfer within BPD: healthy mitochondria or mitochondrial components may be transferred from MSCs, MSC-derived extracellular vesicles/exosomes, or adipose-derived MSC exosomes to injured pulmonary recipient cells, including AEC II, PVEC, and alveolar macrophages, through extracellular vesicles/exosomes or tunneling nanotubes. This transfer can replenish mitochondrial function, restore ATP synthesis, reduce mtROS accumulation, and support epithelial/endothelial survival or macrophage metabolic reprogramming (Omar et al., 2022; Xia et al., 2022). Clinically, intratracheal MSC transplantation has been reported as safe and feasible in preterm infants, and intravenous allogeneic human umbilical cord-derived MSC infusion has shown tolerability in severe BPD, although the efficacy, timing, delivery route, and contribution of mitochondrial transfer still require further validation (Chang et al., 2014; Xia et al., 2022, 2023). Thus, MSC/exosome-based therapy may provide a cell-based or cell-free approach to repairing mitochondrial dysfunction and developmental arrest in BPD.

## 7 Conclusion and perspectives

The discovery of multiple PCD modalities has shifted the understanding of cell demise in BPD from explanations focused solely on apoptosis or necrosis to a model in which mitochondrial dysfunction serves as the initiating event, leading to the coexistence of multiple death pathways. This review proposes that BPD is not merely the result of overactivation of a single cell death pathway, but rather emerges from a “death network” centered on mitochondrial damage as a common upstream hub, which operates through parallel mechanisms across different pulmonary cell types. In AEC II, the mitophagy–apoptosis balance constitutes a key determinant of epithelial fate, while ferroptosis amplifies injury through lipid peroxidation and necroptosis acts as a backup pathway under sustained stress. In PVEC, mitochondrial damage promotes pyroptosis and inflammatory barrier disruption, thereby aggravating vascular developmental arrest. These intracellular decisions and intercellular signaling exchanges are integrated across epithelial, endothelial, immune, and interstitial cells, collectively driving the characteristic pathological outcomes of BPD: alveolar simplification, arrested vascular development, and interstitial fibrosis.

While this review provides a systematic overview of mitochondrial mechanisms and therapeutic strategies in BPD, several limitations should be acknowledged. Primary among these is the focus on mechanistic insights and interventions derived from basic research, with insufficient discussion of their clinical research. Moreover, although multiple cell death pathways and their interactions are described, the review does not adequately address the dynamic spatiotemporal regulation of these processes across different stages of human lung development or among diverse clinical phenotypes of BPD. Therefore, it is essential to move beyond the traditional single-pathway research paradigm and instead view BPD as a disease system characterized by spatiotemporal dynamics. This entails identifying the predominant cell death pathways at different disease stages and, on this basis, developing targeted therapies accordingly.

Looking ahead, future research on BPD should focus on three directions. First, advanced technologies such as single−cell sequencing and spatial transcriptomics should be employed to precisely map the dynamic evolution of mitochondrial injury and cell death signals during hyperoxia exposure, repair, and fibrosis. Second, dynamic intervention strategies need to be developed according to disease progression—focusing primarily on antioxidant/anti−inflammatory approaches in the early stage, promoting mitochondrial biogenesis and reducing the clearance of healthy mitochondria during the repair phase, and targeting anti−fibrosis along with alveolar and pulmonary vascular remodeling in the late stage. Finally, lung organoid models can be utilized for high−throughput screening of multi−target drugs, and delivery systems based on exosomes or mitochondrial transplantation can be developed to enable novel therapies that achieve precise drug enrichment and intervention in the alveolar region. By shifting from a single−pathway perspective to a systemic explanation of disease progression, this approach not only holds promise for overcoming current therapeutic bottlenecks in BPD, but also provides a more universal framework for studying other complex, multi−mechanism diseases in newborns.

## 8 Search strategy

A comprehensive literature search was performed across the electronic databases PubMed, Web of Science, and Embase from their inception through October 2025. The search utilized a combination of Medical Subject Headings (MeSH) terms and keywords related to the core themes of this review. Key terms included, but were not restricted to: “bronchopulmonary dysplasia” OR “BPD” OR “chronic lung disease of prematurity” AND “mitochondria” (encompassing “mitochondrial dysfunction”, “mitochondrial dynamics”, “mitochondrial quality control”) AND “oxidative stress” AND “inflammation” AND “cell death” OR “programmed cell death” (specifically “apoptosis”, “autophagy”, “mitophagy”, “pyroptosis”, “necroptosis”, “ferroptosis”). Boolean operators (AND, OR) were applied to refine the search. The search was confined to articles published in the English language. Both preclinical studies (utilizing animal models and *in vitro* cell cultures) and relevant clinical or translational research were considered for inclusion. To ensure thorough coverage, the reference lists of identified key articles were manually examined to supplement the electronic search with additional pertinent publications. The final selection of literature prioritized seminal works establishing fundamental mechanisms as well as recent studies reporting significant advances in understanding the role of mitochondrial pathophysiology and interlinked cell death pathways in BPD.
